# The influence of shorter red blood cell lifespan on the rate of HbA1c target achieved in type 2 diabetes patients with a HbA1c detection value lower than 7%

**DOI:** 10.1111/1753-0407.13345

**Published:** 2022-12-21

**Authors:** Junmei Wang, Li Zhang, Yu bai, Xinli Wang, Weilin Wang, Jing Li, Saijun Zhou

**Affiliations:** ^1^ NHC Key Laboratory of Hormones and Development Chu Hsien‐I Memorial Hospital and Tianjin Institute of Endocrinology, Tianjin Medical University Tianjin China; ^2^ Tianjin Key Laboratory of Metabolic Diseases Tianjin Medical University Tianjin China

**Keywords:** glycosylated hemoglobin, mean blood glucose, red blood cell lifespan, type 2 diabetes, 糖化血红蛋白, 血糖均值, 红细胞寿命, 2型糖尿病

## Abstract

**Background:**

Variations in the red blood cell (RBC) lifespan can affect glycosylated hemoglobin (HbA1c) test values, but there is still a lack of evidence regarding how and to what degree the RBC lifespan influences HbA1c in the type 2 diabetes mellitus (T2DM) population owing to the restriction of traditional RBC lifespan–detection means. This study aimed to investigate the influence of RBC lifespan variation on HbA1c values in T2DM patients with a HbA1c detection value lower than 7%.

**Methods:**

Patients with HbA1c <7% were divided into two groups: RBC lifespan <90 days and RBC lifespan ≥90 days. We collected blood glucose levels at seven time points for three consecutive months, assessed the HbA1c and glycosylated albumin levels, and calculated the hemoglobin glycation index (HGI) for each patient.

**Results:**

There were no statistical differences in the HbA1c value between two groups, but the estimated glycosylated hemoglobin (eHbA1c) was significantly higher in patients with an RBC lifespan <90 days. The proportion of the eHbA1c ≥7% in the group with an RBC lifespan <90 days was significantly higher than the other group (33.87% vs. 12.50%, *p* < .01). Pearson analysis showed a significant negative correlation between RBC lifespan and the HGI in patients with T2DM (*r* = −0.348, *p* < .01).

**Conclusion:**

A reduced RBC lifespan in T2DM patients caused a noticeable underestimate of the blood glucose levels as presented by HbA1c detection value.

## Graphical abstract

**Highlights**

- Red blood cell (RBC) lifespan variation, is the main cause of variation in glycosylated hemoglobin (HbA1c), independent of blood glucose levels.
- This study highlighted the important impact of RBC lifespan on HbA1c test value in T2DM with HbA1c test value <7%.
- We discovered that the HbA1c test value in type 2 diabetes (T2DM) patients with RBC lifespan <90 days significantly underestimated their general blood glucose condition.
- Our results will provide more useful information to advance management of blood glucose levels for T2DM.

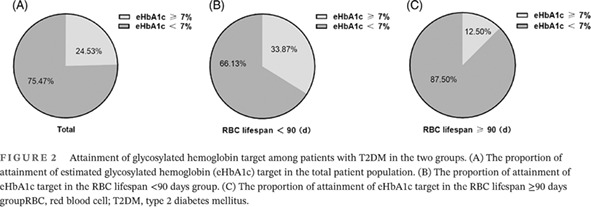

## 摘要

**背景:** 红细胞(RBC)寿命的变化会影响HbA_1c_的检测值, 但由于传统RBC寿命检测手段的限制, 在2型糖尿病(T2DM)人群中, RBC寿命如何以及在多大程度上影响HbA_1c_仍缺乏证据。本研究旨在探讨HbA_1c_检测值低于7%的T2DM患者红细胞寿命变化对HbA_1c_值的影响。

**方法:** 将HbA_1c_<7%的患者分为两组:RBC寿命<90天, 红细胞寿命≥90天。我们连续3个月在7个时间点收集每位患者的血糖水平, 评估糖化血红蛋白(HbA_1c_)和糖化血清白蛋白(GA)水平, 计算糖化血红蛋白指数(HGI)。

**结果:** 两组之间的HbA_1c_值无统计学差异, 但预估糖化血红蛋白值(eHbA_1c_)在RBC<90天寿命的患者中显著升高。红细胞寿命<90天组中, eHbA_1c_≥7%的比例显著高于另一组(33.87% vs 12.50%, p<0.01)。Pearson分析显示, T2DM患者红细胞寿命与HGI呈显著负相关(r =−0.348,p<0.01)。

**结论:** T2DM患者红细胞寿命缩短导致HbA_1c_检测值显示的血糖水平明显被低估。

## 1 INTRODUCTION

With the incidence of type 2 diabetes mellitus (T2DM) rising worldwide, clinical prevention and treatment of T2DM has become a serious global public health problem.^1^ According to the latest epidemiological survey, it was estimated that in 2019 there are 463 million people with diabetes worldwide. The International Diabetes Federation estimated the global prevalence to be 700 million in 2045.^1^ The management of blood glucose in patients with T2DM should be given the top priority, because blood glucose can considerably influence the risk of chronic complications and long‐term prognosis.^2^ Glycosylated hemoglobin (HbA1c) plays a major role in the clinical prevention and treatment of T2DM. HbA1c is one of the most important indices to diagnose T2DM and the main indicator to evaluate whether the blood glucose management of patients with T2DM is up to target. It is also an important basis to guide the clinical adjustment of antihyperglycemic strategy and an important index to judge the clinical prognosis of patients.^2^ Therefore, it is important that the HbA1c test value should accurately reflect the real blood glucose levels in patients with T2DM. However, increasing evidence shows that HbA1c detection value deviated from the general blood glucose condition in patients with T2DM. Hemoglobin glycation variation index (HGI) is an important parameter to evaluate the degree of detection value of HbA1c for average blood glucose.^3^ HGI is the difference between HbA1c and estimated glycosylated hemoglobin (eHbA1c) measured by the date‐matched average blood glucose, which is also gaining increasing attention. Previous study confirmed that HGI is also an independent risk factor for microangiopathy, such as diabetic nephropathy, diabetic retinopathy, and macrovascular disease, in patients with T2DM.^4^, ^5^ The variation in red blood cell (RBC) lifespan is considered to be the main cause of HGI in T2DM patients. The RBC lifespan, referring to the survival time of mature RBCs in the circulating blood after released from the bone marrow, determines the duration of exposure of hemoglobin to glucose. Malka et al reported that differences in RBC longevity can explain nearly 100% of the variation in HbA1c, independent of blood glucose levels.^6^ The RBC lifespan is affected by genetic factors,^7^, ^8^ ethnicity,^9^ and diseases such as hematological diseases,^10^ chronic kidney disease,^11^ hyperglycemia,^12^ and drugs.^13^ Beltran et al verified in a small sample of normal people that the variation of RBC lifespan can cause significant clinical deviation in HbA1c test values.^14^ How much interference does this variation in the RBC lifespan cause to the management of blood glucose of T2DM? There is still a lack of large samples of clinical research data. Therefore, in this study, T2DM patients with a HbA1c detection value <7% (clinically viewed as achieving blood glucose control targets) will be assessed to analyze the relationship between blood glucose levels in patients with different RBC lifespans and HbA1c detection values. Understanding this relationship will aid in the achievement of accurate blood glucose management targets for patients with T2DM.

## 2 MATERIAL AND METHODS

### 2.1 Subjects

We included 326 patients with T2DM who were admitted at the Trinity Care Outpatient Clinic of Chu Hsien‐I Memorial Hospital, Tianjin Medical University (Tianjin, China) from September 2019 to January 2021. Inclusion criteria included adult patients who (1) were >18 years of age and were diagnosed with T2DM based on the 2015 American Diabetes Association diagnostic criteria; (2) received their T2DM diagnosis and at least 3 months of treatment at the Trinity Care clinic; (3) have used a blood glucose monitoring system within the past 3 months; (4) monitor their fasting blood glucose at least twice a week and test their blood glucose via finger stick before each meal and 2 h after each meal, according to the A1C‐derived Average Glucose (ADAG) study^15^; (5) have a glycosylated hemoglobin <7%; (6) have blood glucose levels that have not fluctuated considerably in the past 3 months; (7) have a difference in their maximum and minimum blood glucose levels that is <4.4 mmol/L^16^; and (8) provided their voluntary participation in and cooperation with the study, in addition to a signed informed consent. Exclusion criteria were (1) type 1 diabetes; (2) high blood glucose fluctuation (the difference between the maximum and minimum blood sugar value within a day is >4.4 mmol/L); (3) repeated hypoglycemic attacks (once a week on average); (4) pregnant or lactating women; (5) severe diabetic complications, such as diabetic ketoacidosis, hyperosmotic hyperglycemia syndrome, or lactic acidosis; (6) blood system diseases; (7) malignant neoplasms, tuberculosis, hyperthyroidism, and other chronic expendable diseases (chronic hepatitis, anorexia, chronic colitis, and so on); (8) infectious diseases; (9) chronic liver diseases or alanine aminotransferase (ALT) levels increased more than three times the normal levels; (10) chronic kidney disease (creatinine >1.5 mg/dL); (11) autoimmune diseases; (12) after heart valve replacement or New York Heart Association functional grade III or above; (13) history of blood donation and transfusion within the past 4 months; (14) smoking; (15) impaired lung function; and (16) use of drugs that may affect RBC lifespan, such as ribavirin, barbiturates, or phenobarbital sodium.

This study was approved by the Ethics Committee of Zhu Xianyi Memorial Hospital of Tianjin Medical University (Tianjin, China). All patients provided their written informed consent prior to enrollment in the study.

### 2.2 Data collection and laboratory examination

We collected clinical data, including age, sex, duration of diabetes, and medications, using medical records. Blood glucose values using the iHealth software of the Trinity Care patient management system. The weight, height, and brachial artery blood pressure of the right upper limb of each patient were measured by trained personnel. Fasting venous blood was collected and tested at the central laboratory of Chu Hsien‐I Memorial Hospital, Tianjin Medical University. A Beckman Coulter AU5800 automatic biochemical analyzer (Beckman Coulter, Brea, CA, USA) was used to detect glycosylated serum albumin, liver and kidney function, blood lipids, and other biochemical indices. Each patient's HbA1c levels were tested using a Sysmex XN instrument (Sysmex, Kobe, Japan). Clinical indices were obtained on the same day as that of gas collection.

### 2.3 Mean blood glucose

All of the patients with T2DM enrolled in this study self‐monitored their blood glucose levels using the Trinity Care Outpatient and Diabetes Management System during the previous 3 months. The frequency of blood glucose monitoring followed that of the ADAG study,^15^ and each patient reported their blood glucose level before each meal and 2 h after each meal at least twice a week. The number of blood glucose values per patient was ≥96. The average blood glucose calculation method was based on the ADAG study,^15^ which is the arithmetic average of blood glucose measurement values for each patient. The average blood glucose (AG) can be converted to eHbA1c, eHbA1c = (AG + 2.5944)/1.5944.^17^ HGI refers to the deviation between HbA1c and HbA1c estimated by date‐matched average blood glucose (HGI = HbA1c–eHbA1c).^3^

### 2.4 Carbon monoxide (CO) breath testing and RBC lifespan testing

The RBC detection method was based on a previous study by Ye et al.^18^ Endogenous CO in exhalation breath is mainly derived from the catabolic degradation of heme in hemoglobin released after RBC destruction. Therefore, RBC lifespan at the time of measurement can be estimated on the basis of the total amount of CO that can be released by hemoglobin in the body divided by the amount of CO released by daily hemoglobin degradation. In brief, the alveolar gas samples were collected from each participant after an overnight fast and 20‐min rest. Before sampling, the participant was instructed to take a deep breath, hold it for 10 s, and then exhale into a collection system through the mouthpiece. Simultaneously with the collection of the participant's alveolar gas, the environmental background air sample were collected using an air pump. The ELS Tester (ELS TESTER, Seekya Biotec Co., Ltd., Shenzhen, China), an automated instrument, was used to determine the CO concentrations by nondispersive infrared spectroscopy with paired alveolar and air gas samples, which were used to calculate the RBC lifespan using Levitt's formula.^19^, ^20^ The average RBC lifespan of healthy adults is 115 (70 ~ 140) days.^21^ Previous studies have been based on an RBC lifespan of 90–120 days.^22^ So we considered RBC lifespan <90 days as shorter RBC lifespan group and divided patients into RBC lifespan <90 days group and RBC lifespan ≥90 days group.

### 2.5 Statistical analysis

Statistical analysis was conducted using IBM SPSS 25.0 (IBM Corp., Armonk, NY, USA) and R v 4.0.3 (The R Project for Statistical Computing, Vienna, Austria). A *t* test was used to compare data groups with a normal distribution and a chi‐square test was used to compare data groups with a nonnormal distribution. Two‐sided *p* < .05 values were considered statistically significant.

## 3 RESULTS

### 3.1 Demographic and clinical characteristics of patients with T2DM

Demographic and clinical features of all patients enrolled in this study specifically 326 T2DM patients with HbA1c detection values <7% are shown in Table 1. Among the participants, 165 were men and 161 women, with a mean age of 56.5 ± 11.9 years. The median interquartile range (IQR) duration of T2DM was 5.8 (2, 12) years. The mean blood glucose was 8.07 ± 1.48 mmol/L. The HbA1c was 6.14 ± 0.55%. The 2‐hour postprandial blood glucose (P_2_BG) was 9.20 ± 1.43 mmol/L, and the hemoglobin (Hb) was 142.81 ± 18.6 g/L. There were 183 patients with an RBC lifespan <90 days, and 143 patients with an RBC lifespan ≥90 days. There were no statistical differences in regards to sex, age, systolic blood pressure, diastolic blood pressure, Hb, triglyceride, total cholesterol, low‐density lipoprotein cholesterol, aspartate transaminase, ALT, serum creatinine, urine albumin, blood urea nitrogen, estimated glomerular filtration rate, and therapeutic regimen between the two groups. Metformin was involved in 70.6% of these patients' treatment plans. The duration of T2DM was longer in the RBC lifespan <90 days group than in the RBC lifespan ≥90 days group. The average RBC lifespan of healthy adults is 115 (70 ~ 140) days.^21^ Previous studies have been based on an RBC lifespan of 90–120 days.^22^ In patients with an RBC lifespan <90 days, the average RBC lifespan was 70.0 ± 15.8 days, whereas the average RBC lifespan was 119.8 ± 23.5 days in patients with an RBC lifespan ≥90 days.

**TABLE 1:** General clinical data of patients with T2DM (mean ± SD or median)

Parameters	Total	RBC lifespan <90 days	RBC lifespan ≥90 days	*p* value
*N*	326	183	143	‐
Age (years)	56.5 ± 11.9	55.2 ± 12.1	56.9 ± 11.8	.71
Sex (M/F)	(165/161)	(102/81)	(63/80)	.048
Duration (years)	5.8 (2, 12)	7 (3, 13)	4.3 (2, 10)	<.01
SBP (mm Hg)	135.6 ± 17.4	136.2 ± 17.9	134.9 ± 16.7	.53
DBP (mm Hg)	80.4 ± 9.6	80.6 ± 9.2	80.1 ± 10.2	.60
Hb (g/L)	142.2 ± 18.6	144.1 ± 20.4	143.5 ± 15.1	.14
AG (mmol/L)	8.07 ± 1.48	8.30 ± 1.50	7.74 ± 1.06	<.0001
FBG (mmol/L)	7.20 ± 1.47	7.41 ± 1.76	6.88 ± 0.92	<.01
P2BG (mmol/L)	9.20 ± 1.43	9.61 ± 2.12	8.80 ± 1.34	<.001
TG (mmol/L)	1.72 ± 1.02	1.76 ± 1.13	1.66 ± 0.85	.37
TC (mmol/L)	4.76 ± 1.13	4.65 ± 1.22	4.87 ± 0.98	.08
LDL‐c (mmol/L)	3.33 ± 2.43	3.21 ± 0.98	3.47 ± 2.83	.32
ALT (U/L)	22.9 ± 13.6	23.3 ± 13.0	22.2 ± 14.2	.46
AST (U/L)	22.5 ± 8.8	22.9 ± 9.8	21.8 ± 7.3	.27
SCr (μmol/L)	69.3 ± 23.8	73.0 ± 23.5	64.7 ± 17.2	.12
BUN (μmol/L)	5.59 ± 2.24	5.86 ± 2.71	5.26 ± 1.41	.06
UA (μmol/L)	345.3 ± 91.2	346.9 ± 89.1	343.1 ± 93.7	.71
eGFR (ml/min)	93.6 ± 20.0	91.4 ± 20.7	96.2 ± 18.7	.07
Insulin only (%)	2.8	2.2	3.5	.48
Combination of insulin and other hypoglycemic drugs (%)	23.9	20.3	19.6	.10
Without insulin (%)	73.3	70.5	76.9	.25

*Note*: *p* value: RBC lifespan <90 days versus, RBC lifespan ≥90 days.

Abbreviations: AG, average blood glucose; ALT, alanine aminotransferase; AST, aspartate transaminase; BUN, blood urea nitrogen; DBP, diastolic blood pressure; eGFR, estimated glomerular filtration rate; FBG, fasting blood glucose; Hb, hemoglobin; LDL‐c, low‐density lipoprotein cholesterol; P_2_BG, 2‐hour postprandial blood glucose; RBC, red blood cell; SBP, systolic blood pressure; SCr, serum creatinine; T2DM, type 2 diabetes mellitus; TC, total cholesterol; TG, triglyceride; UA, urine albumin.

### 3.2 Comparison of blood glucose in the two groups of patients with T2DM

**FIGURE 1:**
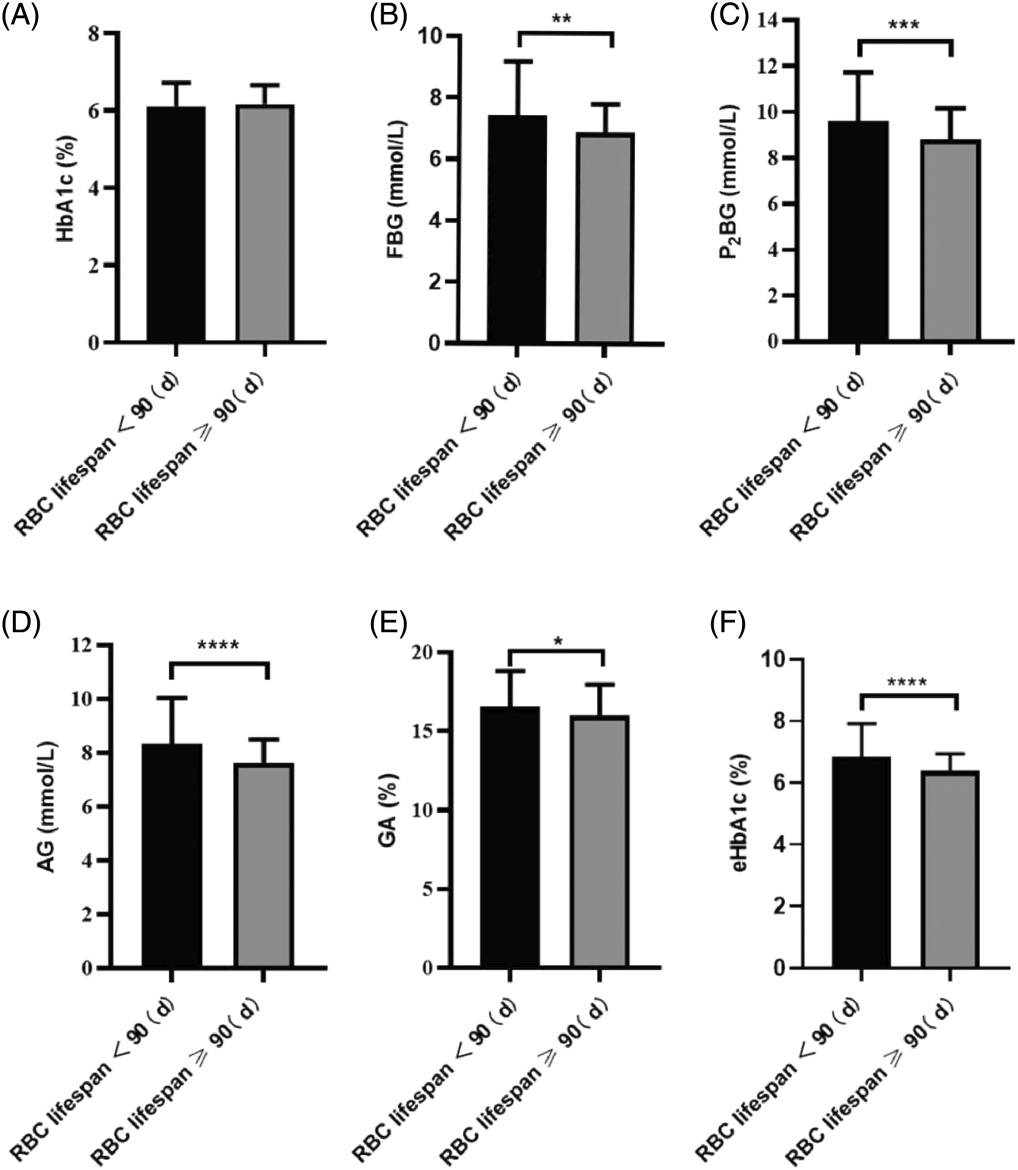
Comparison of blood glucose levels in the two groups of patients with T2DM. (A) Comparison of glycosylated hemoglobin (HbA1c) in the two groups. (B) Comparison of fasting blood glucose (FBG) in the two groups. (C) Comparison of 2‐h postprandial blood glucose (P_2_BG) in the two groups. (D) Comparison of average blood glucose (AG) in the two groups. (E) Comparison of glycosylated albumin (GA) in the two groups. (F) Comparison of estimated glycosylated hemoglobin (eHbA1c) in the two groups, eHbA1c = (AG + 2.5944)/1.5944. *p < .05; **p < .01;***p < .001; ****p < .0001. RBC, red blood cell; T2DM, type 2 diabetes mellitus.

As shown in Figure 1, the study began by comparing blood glucose control in patients with T2DM during the previous 3 months. Although we found no statistical difference in HbA1c levels between the two groups, the blood glucose levels were significantly higher in the group with an RBC lifespan <90 days as compared to the group with an RBC lifespan ≥90 days. Additionally, the levels of fasting blood glucose (FBG), P_2_BG, AG, and glycosylated albumin (GA) in the group with an RBC lifespan <90 days were significantly higher as compared to the group with an RBC lifespan ≥90 days. We also confirmed that the HbA1c test values were significantly underestimated in patients with an RBC lifespan <90 days. This study calculated the eHbA1c levels of the two groups based on the blood glucose levels of the patients, and as is shown in Figure 1F, the levels of eHbA1c in the RBC lifespan <90 days group were significantly higher as compared to the RBC lifespan ≥90 days group. These results indicated that shortening the RBC lifespan in patients with T2DM may affect the patient's blood glucose target attainment.

### 3.3 eHbA1c target attainment among patients with T2DM in the two groups

**FIGURE 2:**
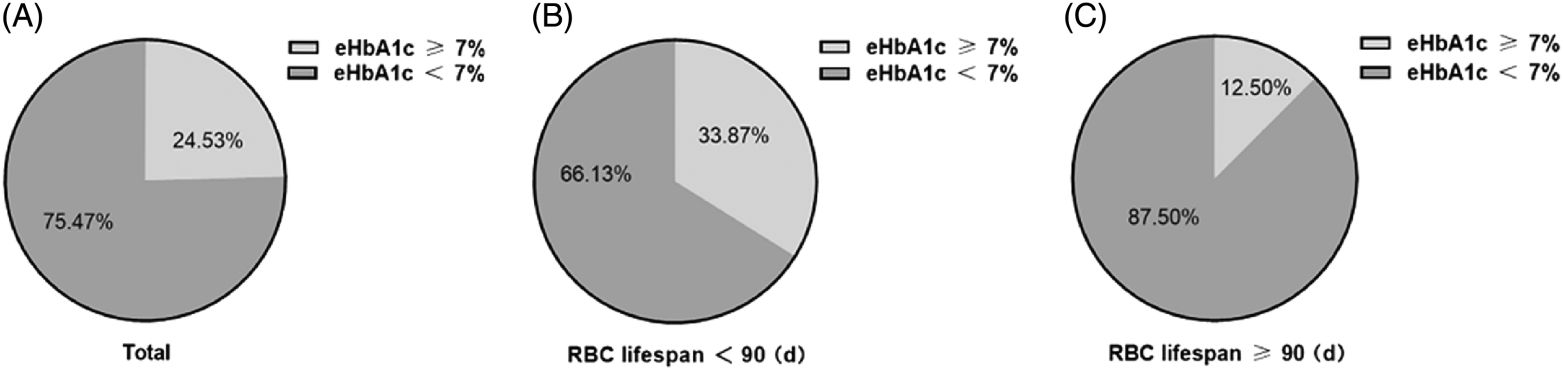
Attainment of glycosylated hemoglobin target among patients with T2DM in the two groups. (A) The proportion of attainment of estimated glycosylated hemoglobin (eHbA1c) target in the total patient population. (B) The proportion of attainment of eHbA1c target in the RBC lifespan <90 days group. (C) The proportion of attainment of eHbA1c target in the RBC lifespan ≥90 days groupRBC, red blood cell; T2DM, type 2 diabetes mellitus.

To confirm that the HbA1c detection value led to an underestimation of the blood glucose in patients in the RBC lifespan <90 days group, we further evaluated the target attainment of HbA1c in the two groups according to the patients' eHbA1c level. We set an eHbA1c value <7% as the target for glycated hemoglobin. As shown in Figure 2A, among the 326 patients with HbA1c detection values <7%, 24.53% had an eHbA1c ≥ 7%. As is shown in Figure 2B,C, 33.87% of the patients in the RBC lifespan <90 days group had eHbA1c values ≥7%, which was significantly higher than that seen in patients in the RBC lifespan ≥90 days group (12.50%). These result suggested that a shortened RBC lifespan fails to accurately reflect the patients' blood glucose level, resulting in poor blood glucose control.

### 3.4 The pattern of the effect of shortened RBC lifespan on HbA1c test value

**FIGURE 3:**
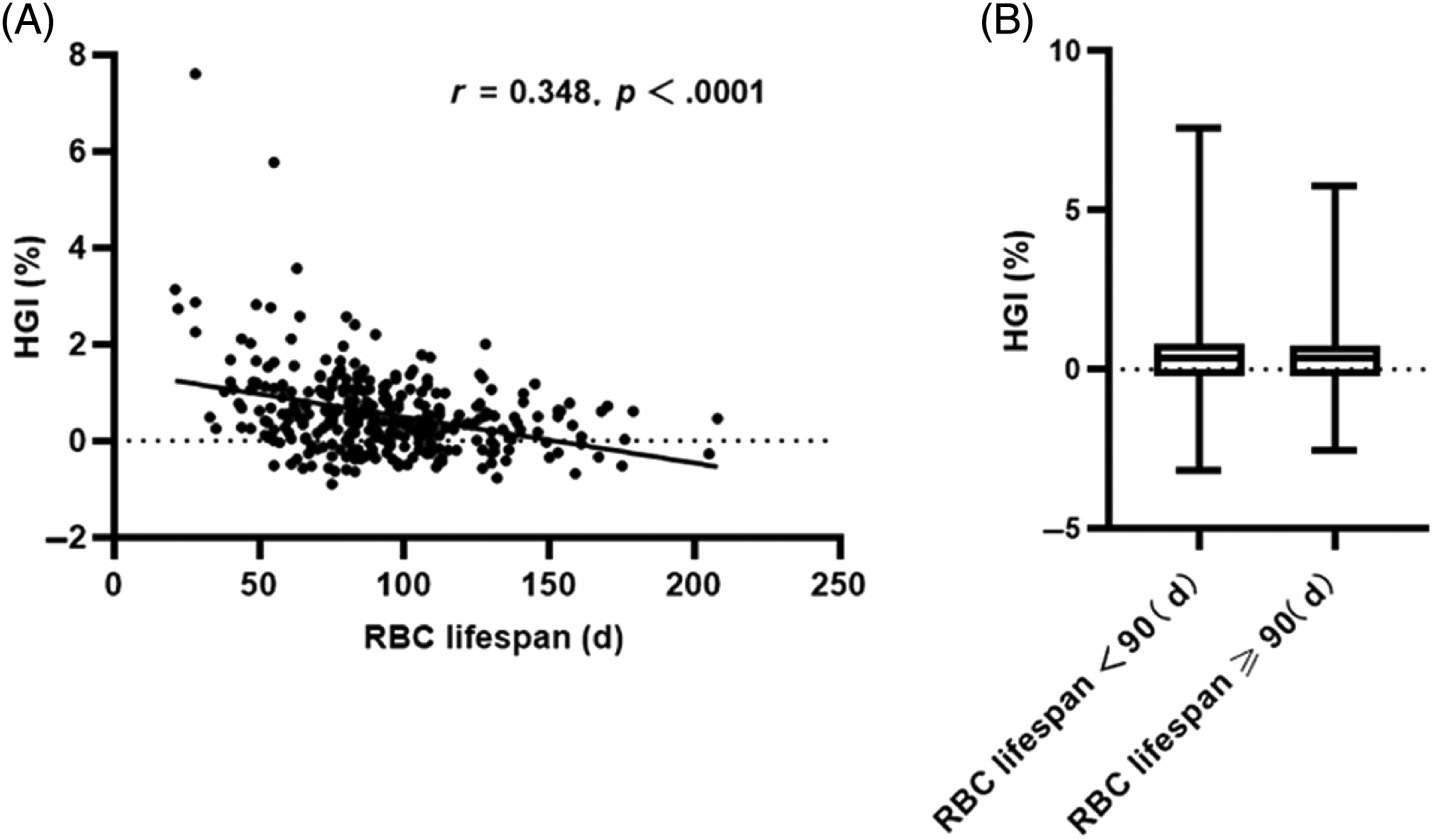
Relationship between RBC lifespan and hemoglobin glycation index (HGI) in patients with type 2 diabetes mellitus (T2DM). Pearson correlation analysis of RBC lifespan and HGI in patients with T2DM, HGI = eHbA1c‐HbA1c. (B) Comparison of HGI in the two groupseHbA1c, estimated glycosylated hemoglobin; RBC, red blood cell.

The HGI is an important indicator of whether HbA1c measurement values deviate from a patient's average blood glucose.^16^ We first analyzed the relationship between RBC lifespan and HGI in patients with T2DM using a Pearson correlation analysis. As shown in Figure 3A, there was a significant negative correlation between RBC lifespan and HGI. The median HGI (IQR) was 0.58% (0.18, 1.11) in the RBC lifespan <90 days group, and the median HGI (IQR) was 0.25% (−0.09, 0.55) in the RBC lifespan ≥90 days group. The results of this study confirmed that a shortened RBC lifespan can lead to an underestimation of HbA1c values in patients with T2DM.

## 4 DISCUSSION

HbA1c is an important index not only to diagnose T2DM and evaluate the blood glucose management of T2DM patients but also to guide both the antihyperglycemic therapy and prognosis of T2DM patients. Because the HbA1c detection value is known to be significantly influenced by RBC lifespan, it is critical that the influence of RBC lifespan on blood glucose levels in patients with T2DM be understood. This study was conducted in T2DM patients who were clinically assessed to meet the target of glycemic management (ie, HbA1c test values <7%) to compare the difference in blood glucose levels among patients with differing RBC lifespans.

In this study, patients with T2DM were divided into two groups according to RBC lifespan: RBC lifespan <90 days and RBC lifespan ≥90 days group. There is growing evidence that HbA1c levels depend not only on average blood glucose levels but also on RBC lifespan.^23^ Using radioactive iron‐labeled RBCs, a previous study tracked the cell age of mice and found that HbA1c levels increased over the lifetime of the RBCs.^24^ This study confirmed that HbA1c levels were affected by the lifespan of RBCs in T2DM patients. In our study, we found no statistical differences in HbA1c between the two groups. Therefore, the blood glucose levels of the two groups of patients were considered to not be statistically different. The levels of FBG, P_2_BG, mean blood glucose, GA, and eHbA1c were significantly higher in the group with an RBC lifespan <90 days as compared to the group with an RBC lifespan ≥90 days. This study calculated the eHbA1c levels of the two groups based on blood glucose levels of the patients and found that the levels of eHbA1c in the group with the RBC lifespan <90 days was significantly higher than that of the group with the RBC lifespan ≥90 days (Figure 1F). According to the results, a shortened RBC lifespan resulted in the HbA1c detection value to be underestimated, which led to less intensification of treatment or poor compliance. Our results indicated that the duration of T2DM was longer in the RBC lifespan <90 days group than in the RBC lifespan ≥90 days group. The reason could be hyperglycemia in diabetic patients causing the shortening of RBC lifespan and T2DM patients with long‐term hyperglycemia may be more likely to experience the RBC lifespan shortening. The duration of T2DM was longer in the group with RBS lifespan <90 days, which can also confirm that the actual blood glucose level in this group is higher than the other group.

To analyze the influence of a shortened RBC lifespan on the target attainment of the HbA1c detection value in T2DM patients, this study compared the eHbA1c values between the two groups. As shown in Figure 2, among the 326 T2DM patients, clinically viewed as reaching HbA1c targets (with HbA1c detection values <7%), the proportion of patients for whom the eHbA1c ≥7% was 24.53%, and the eHbA1c ≥7% in the RBC lifespan <90 days and lifespan ≥90 days group was 33.87% and 12.50% respectively. This result strongly indicates that shorter RBC lifespans give rise to a considerable number of T2DM patients' underestimated HbA1c values, which results in their failing to meet their blood glucose management target. The results of this study might explain, in part, the early onset of diabetic nephropathy and diabetic retinopathy in T2DM patients according to the clinical standard.^4^ Why does a shorter RBC lifespan lead to the underestimation of the HbA1c detection value? The reaction of glycation hemoglobin is the proportion of hemoglobin being glycosylated, which is exposed to certain blood sugar level during the survival time of RBCs. The variation in RBC lifespan determines the duration of exposure of hemoglobin to glucose. As a result, a shorter RBC lifespan leads to the shorter time of hemoglobin being glycosylated, which influences the total synthesis of HbA1c. Meanwhile, Malka et al indicated that between‐patient variation in derived mean RBC age explains all glucose‐independent variations in HbA1c,^6^ and Kameyama et al showed the hyperbolic relationship between AG and HbA1c based on a precise RBC lifespan distribution using a good‐fitted RBC lifespan model, which also indicated that the differences in HbA1c for a given mean blood glucose value can be explained by the average age of RBC lifespan.^25^ So the variation in the RBC lifespan of patients with T2DM was an important reason for the deviation of HbA1c detection values from the average blood glucose levels.

In recent years, the deviation between HbA1c and HbA1c estimated by date‐matched average blood glucose, namely, HGI, has led some diabetes professionals to question the accuracy of HbA1c. Studies have confirmed that HGI is an independent risk factor for microangiopathy, such as diabetic nephropathy, diabetic retinopathy, and macrovascular disease, in patients with T2DM.^4^, ^5^ This was because the HbA1c values of patients with a large HGI do not accurately reflect the patients' blood glucose level, resulting in less intensification of treatment or poor compliance. Pearson analysis showed a significant correlation between RBC lifespan and HGI in T2DM (Figure 3A). The levels of HGI in the group with an RBC lifespan <90 days was significantly higher than that of the group with an RBC lifespan ≥90 days. The results of this study suggested that the variation in the RBC lifespan in patients with T2DM influences the HGI and also suggested further thinking to more accurately evaluate the HbA1c value by detecting RBC lifespan.

Limitations of this study include relatively small sample size, average glucose not derived by continuous glucose monitoring, the effect of the background level of CO in the sampling location on RBC lifespan detection values, and the difficulty in correcting the impact of shorter RBC lifespan.

In conclusion, the variation in the RBC lifespan of patients with T2DM was an important reason for the deviation of HbA1c detection values from the average blood glucose levels, that is, the HGI. A considerable proportion of patients with T2DM and a shortened RBC lifespan had significantly underestimated HbA1c detection values and blood glucose levels. Thus, accurate evaluation of the blood glucose level in patients with T2DM with shortened RBC lifespan is important, and the effect of RBC lifespan on HbA1c values must be considered. Our results will provide more useful information about blood glucose management for clinical practice and improve patients' therapy compliance.

## AUTHOR CONTRIBUTIONS

Saijun Zhou conceived the project and designed experiments. Junmei Wang and Li Zhang collected the patients' information and analyzed data. Yu Bai, Junmei Wang, Li Zhang, Xinli Wang, Weilin Wang, and Jing Li collected the patients' information. Junmei Wang and Saijun Zhou had input in writing the paper.

## DISCLOSURE

None declared.
